# Acute dosing of latrepirdine (Dimebon™), a possible Alzheimer therapeutic, elevates extracellular amyloid-β levels *in vitro *and *in vivo*

**DOI:** 10.1186/1750-1326-4-51

**Published:** 2009-12-17

**Authors:** John W Steele, Soong H Kim, John R Cirrito, Deborah K Verges, Jessica L Restivo, David Westaway, Paul Fraser, Peter St George Hyslop, Mary Sano, Ilya Bezprozvanny, Michelle E Ehrlich, David M Holtzman, Sam Gandy

**Affiliations:** 1 Departments of Neurology, Psychiatry and Alzheimer's Disease Research Center, Mount Sinai School of Medicine, New York, NY, 10029, USA; 2 Department of Neurology, Hope Center for Neurological Disorders and Alzheimer's Disease Research Center, Washington University School of Medicine, St Louis, MO, 63110, USA; 3 Centre for Prions and Protein Folding Diseases, University of Alberta, Edmonton, AB, T6G 2M8, Canada; 4 Centre for Research in Neurodegenerative Diseases, University of Toronto, Toronto, ON, M5S 3H2, Canada; 5 Department of Clinical Neurosciences, University of Cambridge, Cambridge, CB2 0XY, UK; 6 Department of Psychiatry, and Alzheimer's Disease Research Center, Mount Sinai School of Medicine, New York, NY, 10029, USA; 7 Department of Neurology, James J Peters VA Medical Center, Bronx, NY, 10468 USA; 8 Department of Physiology, University of Texas Southwestern, Dallas, TX, 75390, USA; 9 Departments of Pediatrics and Neurology, and Alzheimer's Disease Research Center, Mount Sinai School of Medicine, New York, NY, 10029, USA

## Abstract

**Background:**

Recent reports suggest that latrepirdine (Dimebon™, dimebolin), a retired Russian antihistamine, improves cognitive function in aged rodents and in patients with mild to moderate Alzheimer's disease (AD). However, the mechanism(s) underlying this benefit remain elusive. AD is characterized by extracellular accumulation of the amyloid-β (Aβ) peptide in the brain, and Aβ-lowering drugs are currently among the most popular anti-amyloid agents under development for the treatment of AD. In the current study, we assessed the effect of acute dosing of latrepirdine on levels of extracellular Aβ using *in vitro *and *in vivo *experimental systems.

**Results:**

We evaluated extracellular levels of Aβ in three experimental systems, under basal conditions and after treatment with latrepirdine. Mouse N2a neuroblastoma cells overexpressing Swedish APP were incubated for 6 hr in the presence of either vehicle or vehicle + latrepirdine (500pM-5 μM). Synaptoneurosomes were isolated from TgCRND8 mutant APP-overexpressing transgenic mice and incubated for 0 to 10 min in the absence or presence of latrepirdine (1 μM or 10 μM). Drug-naïve Tg2576 Swedish mutant APP overexpressing transgenic mice received a single intraperitoneal injection of either vehicle or vehicle + latrepirdine (3.5 mg/kg). Picomolar to nanomolar concentrations of acutely administered latrepirdine increased the extracellular concentration of Aβ in the conditioned media from Swedish mutant APP-overexpressing N2a cells by up to 64% (p = 0.01), while a clinically relevant acute dose of latrepirdine administered i.p. led to an increase in the interstitial fluid of freely moving APP transgenic mice by up to 40% (p = 0.01). Reconstitution of membrane protein trafficking and processing is frequently inefficient, and, consistent with this interpretation, latrepirdine treatment of isolated TgCRND8 synaptoneurosomes involved higher concentrations of drug (1-10 μM) and led to more modest increases in extracellular Aβ_x-42 _levels (+10%; p = 0.001); of note, however, was the observation that extracellular Aβ_x-40 _levels did not change.

**Conclusions:**

Here, we report the surprising association of acute latrepirdine dosing with elevated levels of extracellular Aβ as measured in three independent neuron-related or neuron-derived systems, including the hippocampus of freely moving Tg2576 mice. Given the reported association of chronic latrepirdine treatment with improvement in cognitive function, the effects of chronic latrepirdine treatment on extracellular Aβ levels must now be determined.

## Background

Alzheimer disease (AD), the major cause of late-life dementia, is neuropathologically distinguished by the accumulation in brain of extracellular protein deposits of the amyloid-β (Aβ) peptide. A large body of evidence suggests that the major underpinnings of the cognitive decline associated with AD are synaptic and neuronal loss and tauopathy that occur in association with aggregation of Aβ peptide to form a range of structures including high-molecular weight soluble oligomers [1-6]. Most studies have focused on extracellular accumulation of Aβ, although aggregation of the peptide inside intracellular [7-9] transport vesicles has also been described. Current formulation of the "amyloid hypothesis of AD" is that aggregated forms of Aβ, which build up in the human brain over 10-20 years while individuals are still cognitively intact, lead to slowly progressive neurodegeneration and dementia [10]. Hence, drug discovery programs aimed at lowering extracellular Aβ levels have become mainstream in many academic and pharmaceutical AD therapeutics units [5]. Recently, in a double-blind, placebo-controlled clinical trial, the retired Russian antihistamine latrepirdine (Dimebon™, dimebolin) improved and stabilized cognitive function of patients with mild to moderate AD over a one year period [11]. In addition, latrepirdine demonstrated a small benefit in cognitive function in Huntington's disease (HD) [12].

In laboratory studies, latrepirdine has also been associated with an improvement in learning behavior in the rat AF64A model of acetylcholine deficiency [13,14]. A screen against a panel of biochemical targets *in vitro *indicated that latrepirdine demonstrates a number of bioactivities, including modulation of Ca^2+ ^flux [15] and apoptosis, as well as >90% inhibition of α-adrenergic receptors (α_1A_, α_1B_, α_1D_, α_2A_), histamine receptors (H_1 _and H_2_), and serotonin receptors (5-HT_2C_, 5-HT_5A_, 5-HT_6_) at 10 μM concentration [16]. Latrepirdine also potentiates the activity of AMPA receptors in rat cortical and cerebral Purkinje neurons at low concentrations and blocks NMDA receptor-induced currents at higher concentrations [17]. A role in mitochondrial stabilization has also been suggested [18]. However, it is not yet known whether the observed clinical benefits to AD or HD patients are related to any of these effects or to as-yet undiscovered mechanism(s).

Neurotransmitters [19-22], hormones [19,20,23,24], and mitochondrial actions are also associated with changes in Aβ metabolism [20]. Based on this knowledge and on the drug's possible clinical benefit, we investigated whether latrepirdine regulates Aβ levels in *in vitro *and *in vivo *experimental systems. We observed that acute administration of clinically relevant levels of latrepirdine stimulated extracellular accumulation of Aβ in three extracellular systems, namely: (1) the conditioned media of cultured human Swedish mutant (Swe)APP-transfected mouse neuroblastoma cells, (2) the releasate of cortical synaptoneurosomes isolated from TgCRND8 (Swe/Indiana APP) transgenic mice, and (3) the interstitial fluid from the brains of freely moving Tg2576 SweAPP transgenic mice.

## Results

### Latrepirdine increases extracellular Aβ levels in conditioned media of cultured cells

**Figure 1:**
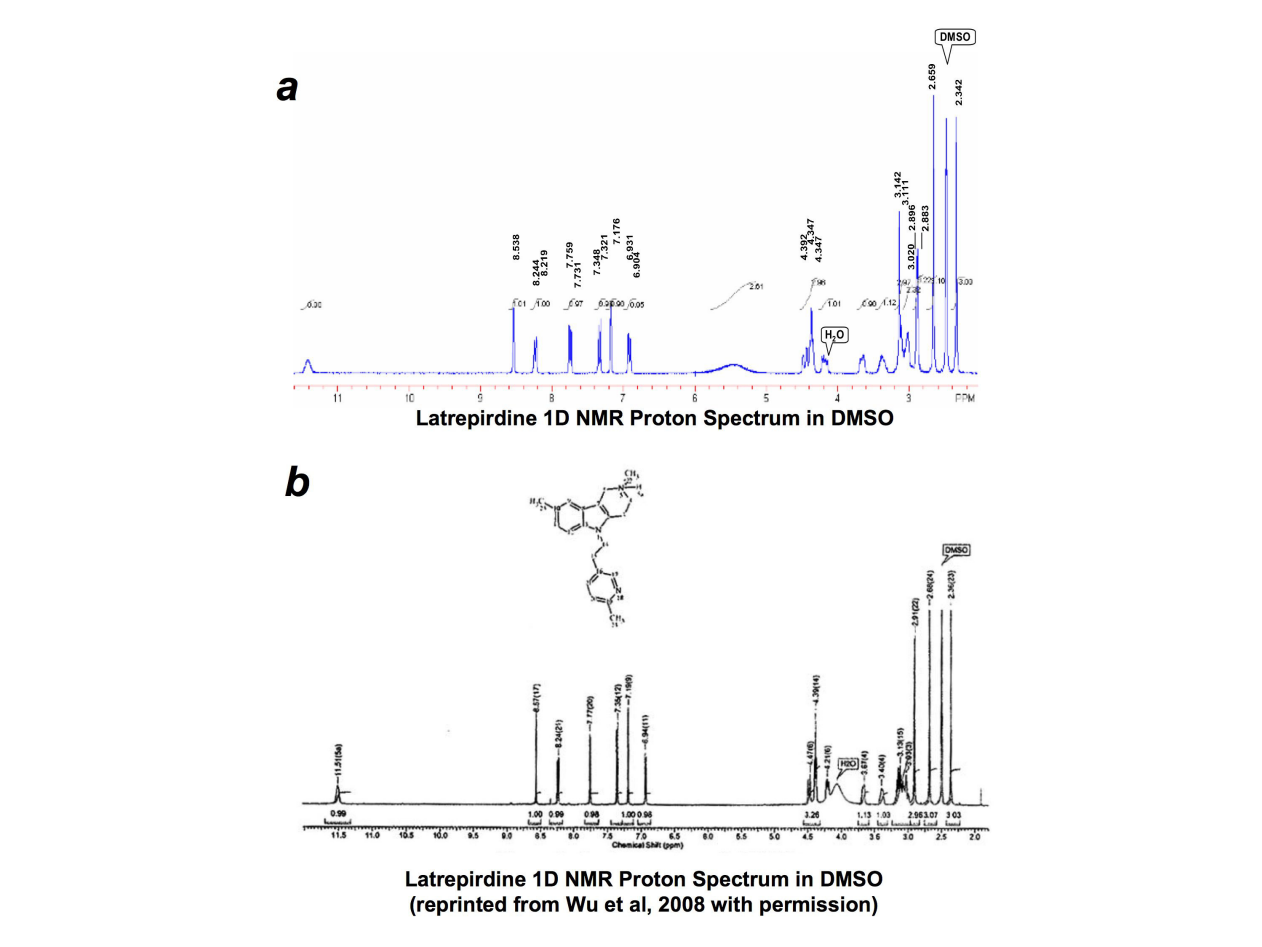
**Comparison of latrepirdine 1D NMR proton spectra in DMSO from two sources**. Latrepirdine was custom synthesized by either Nanosyn Inc or SinoChemexper and purity was determined to be ~97.3% or >99.0%, respectively. 1D NMR proton spectra are compared here for latrepirdine synthesized by SinoChemexper (top) or Nanosyn (bottom, reprinted with permission from Wu et al, 2008 [16]). Aliquots of latrepirdine from both sources were tested in each protocol. Identical effects were observed regardless of source of compound, and data were pooled for overall analysis.

**Figure 2:**
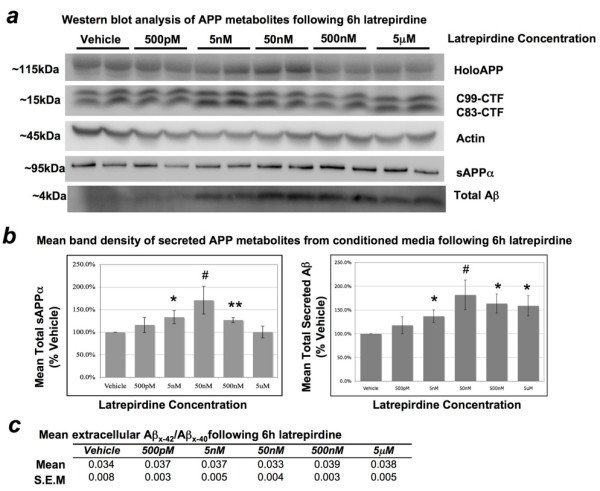
**Effect of latrepirdine on the levels of APP metabolites from cell lysates and conditioned media of mouse N2a cells**. Cells were treated for 6 hours in the presence of vehicle or increasing concentrations of latrepirdine (as labeled). (*a*) Representative western blot of total intracellular and secreted metabolites of APP from at least 3 independent experiments, each performed in duplicate. (*b*) Quantification of western blot band densitometry represented as mean percent of vehicle +/- S.E.M. 5 nM latrepirdine produced an approximately half-maximal ~37% increase (*SD = *0.33, t(10) = 2.75, *p = *0.02), and 500 nM produced a maximal significant ~64% increase (*SD = *0.49, t(10) = 3.16, *p = *0.01) in extracellular Aβ compared to vehicle. The highest concentration (5 μM) also caused a significant ~59% increase (*SD *= 0.52, t(10) = 2.78, *p = *0.02) in extracellular Aβ levels, and an increase in extracellular Aβ levels was observed with 50 nM latrepirdine, which approached significance on a two-tailed test (*SD *= 0.54, t(4) = 2.62, *p *= 0.059). Incubation with 5 nM or 500 nM latrepirdine resulted in a significant ~34% (*SD *= 0.35, t(10) = 2.33, *p *= 0.04) and ~27% (*SD *= 0.14, t(10) = 4.87, *p *= 0.0006) increase in sAPPα accumulation, respectively, in conditioned media compared to vehicle. An increase in extracellular sAPPα levels was observed with 50 nM latrepirdine, which approached significance on a two-tailed test (*SD *= 0.53, t(4) = 2.31, *p *= 0.081). No significant increases in sAPPα levels were distinguished between vehicle and either 500 pM or 5 μM latrepirdine. No significant accumulation of holoAPP of APP-CTFs (C83-CTF or C99-CTF) was observed following 6 h incubation in the absence (vehicle) or presence of varying concentrations of latrepirdine (as indicated). (*c*) Vehicle and all latrepirdine concentrations were indistinguishable by mean Aβ_x-42_/Aβ_x-40 _ratio, quantified by Aβ species-specific sandwich ELISA (mean Aβ_x-42_/Aβ_x-40_, *n *= 3 independent experiments, each performed in duplicate). Absolute Aβ_x-40 _and Aβ_x-42 _levels in the media were ~380-490 pM and ~14-20 pM, respectively. *Value represents a significant mean difference from vehicle by independent samples t-test, two-tailed α = 0.05, #p < 0.10; *p < 0.05; **p < 0.01.

To examine the effects of latrepirdine on Aβ generation *in vitro*, we treated neuroblastoma (N2a) cells stably overexpressing Swedish APP [25] for 6 hours either in the absence (vehicle) or presence of latrepirdine (ranging from 500 pM to 5 μM, as indicated). Latrepirdine was custom synthesized by two independent sources. Both compounds were used for each experiment (Figure 1), and no differences were observed between the two compounds. Cells treated with latrepirdine showed an ~30-64% increase in total extracellular Aβ, as determined by western blot analysis (Figures 2a, b). The effect of latrepirdine on extracellular levels of Aβ appeared to be concentration-dependent, where the lowest concentration (500 pM) produced no significant increase, 5 nM produced an approximately half-maximal ~37% increase and 500 nM produced a maximal significant ~64% increase in extracellular Aβ as compared to vehicle-treated cells (Figure 2, *p *= 0.02 and *p *= 0.01, respectively). The highest concentration (5 μM) also caused a significant ~59% increase in extracellular Aβ levels (Figure 2; *p *= 0.02). These data appear to represent a concentration-dependent effect of latrepirdine with a maximal significant response at 500 nM (see Figure 2).

The amyloid precursor protein (APP) is processed by divergent proteolytic pathways. In the predominant pathway, APP is first cleaved by the α-secretase in the middle of the Aβ domain, generating a large secreted N-terminal ectodomain (sAPPα) and an 83 amino acid membrane-bound C-terminal fragment (CTF; C83-CTF). In another, amyloidogenic, pathway APP is cleaved first by the β-secretase, generating a smaller secreted N-terminal ectodomain (sAPPβ) and a larger 99 amino acid membrane-bound C-terminal fragment (C99-CTF). The γ-secretase complex (PS1, APH-2, PEN-1, Nicastrin) then processes the CTF to generate either the p3 (from C83-CTF) or Aβ (from C99-CTF) peptide of varying lengths (i.e. Aβ varies from 37-44 amino acids in length following γ-secretase cleavage). A significant increase in secretion of sAPPα was also observed following 6 h incubation of cultured N2a cells with 5 nM (~33% increase, *p *= 0.04) and 500 nM (~27% increase, *p *= 0.0006) in comparison to vehicle, but not with either 500 pM or 5 μM in comparison to vehicle (Figure 2). The effects of latrepirdine on extracellular Aβ accumulation may be due to increased overall metabolism of APP, indicative of stimulation of APP trafficking to the plasma membrane. Therefore, we further investigated whether latrepirdine affects processing of holoAPP or its membrane bound CTFs, however no significant differences in holoAPP, C83, or C99 levels were observed in comparison to vehicle (Figure 2a). A small and not significant, but clear increase in accumulation of APP-CTFs and holoAPP was associated with increased extracellular accumulation of sAPPα and Aβ following incubation with 5 nM or 50 nM latrepirdine, suggesting overall increases in APP synthesis and metabolism.

Aβ_x-42_/Aβ_x-40 _ratio has been reported to determine propensity for amyloid deposition, perhaps because Aβ_x-40 _antagonizes the tendency of Aβ_x-42 _to aggregate [26]. Therefore, conditioned media from the same experiments were also analyzed for Aβ_x-42_/Aβ_x-40 _ratio by sandwich ELISA. No significant differences in Aβ_x-42_/Aβ_x-40 _ratio were observed between vehicle and any concentration of latrepirdine tested (Aβ_x-42_/Aβ_x-40 _was ~1:25 for all conditions, Figure 2c). Accumulation of APP-CTFs is often associated with inhibition of the γ-secretase complex cleavage of their membrane-bound substrates and changes in Aβ_x-42_/Aβ_x-40 _ratio is also often indicative of altered γ-secretase activity. Here, we observed neither accumulation of APP-CTFs nor altered Aβ_x-42_/Aβ_x-40 _ratio, strongly suggesting that latrepirdine does not regulate γ-secretase activity in N2a cells.

### Latrepirdine stimulates acute secretion of Aβ_x-42 _from isolated cortical synaptoneurosomes

**Figure 3:**
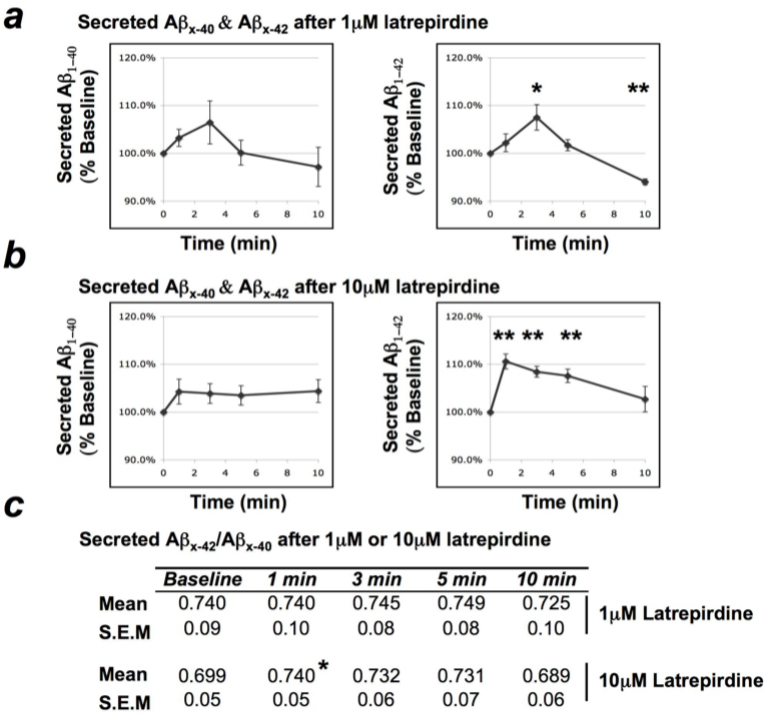
**Secreted Aβ_x-40 _and Aβ_x-42 _levels in the releasates of synaptoneurosomes following incubation with latrepirdine**. Post-natal day 10-14 TgCRND8 cortical synaptoneurosomes were incubated 0 (baseline), 1, 3, 5, or 10 minutes in the presence of 1 μM (n = 5) or 10 μM (n = 6) latrepirdine. (*a*) 1 μM latrepirdine stimulates an increase in secretion of Aβ_x-42_, but not Aβ_x-40_, from isolated synaptoneurosomes following 3 minutes (*SD *= 0.06, t(4) = 2.81, *p = *0.048) of incubation with the drug, and a decrease (~6%) is observed at 10 minutes (*SD *= 0.01, t(5) = 9.61, *p = *0.0007), likely representing a depletion of available Aβ_x-42 _(mean % baseline +/- S.E.M). *(b) *10 μM latrepirdine stimulates an increase in secretion of Aβ_x-42_, but not Aβ_x-40_, following 1 (*SD = *0.038, *t(5) = 6.73, p = *0.001), 3 (SD = 0.028, t(5) = 7.35, *p = *0.0007), and 5 (*SD *= 0.056, t(5) = 5.29, *p = *0.0029) minutes of incubation with the drug (mean % baseline +/- S.E.M). *(c) *An immediate ~4.1% increase (*SD = *0.13, t(5) = 3.205, *p *= 0.024) in Aβ_x-42_/Aβ_x-40 _ratio was observed following 1 minute of incubation with 10 μM latrepirdine (mean Aβ_x-42_/Aβ_x-40_). (Note: TgCRND8 mice generate ~50%/50% Aβ_x-42_/Aβ_x-40 _under normal conditions and the increase in Aβ_x-42_/Aβ_x-40 _ratio may be due to the large stimulation of Aβ secretion at this time point). *Value represents a significant mean difference between baseline and time-point by paired t-test, two-tailed α = 0.05, *p < 0.05, **p < 0.01.

TgCRND8 mice encode a double mutant form of human APP, harboring both Swedish and Indiana mutations under the control of the PrP gene promoter, resulting in a significantly increased Aβ_x-42_/Aβ_x-40 _ratio [27]. A recent report indicated that synaptic activity reduces intraneuronal Aβ by promoting APP transport to synapses and secretion of Aβ into extracellular space, protecting against Aβ-related synaptic alterations [28]. Based on a brief size-selection of synaptic particles, isolated cortical synaptoneurosome preparations from P10-P14 TgCRND8 mice provide an intact, synapse-specific [29] method for the study of nerve terminal biology that we have applied to the study of signal-mediated generation and secretion of Aβ. The releasates from synaptoneurosomes were collected at 0 (baseline), 1, 3, 5, and 10 minutes following incubation with either 1 μM or 10 μM latrepirdine and analyzed for Aβ_x-42 _and Aβ_x-40 _levels by sandwich ELISA. A paired samples t-test was used to compare mean Aβ_x-42 _or Aβ_x-40 _levels for each post-treatment time point versus baseline for each concentration of latrepirdine. Treatment of synaptoneurosomes with 1 μM (Figure 3a) or 10 μM (Figure 3b) latrepirdine resulted in increased secretion of Aβ_x-42_, but not Aβ_x-40_. A 10% increase of secreted Aβ_x-42_, but not Aβ_x-40_, was observed at 1 minute following incubation with 10 μM latrepirdine (p = 0.001). Similar increases were observed at 3 minutes and 5 minutes following stimulation (8% increase at 3 minutes, p = 0.0007; 7.6% increase at 5 minutes, *p = *0.0029, respectively). Latrepirdine at 1 μM stimulated a significant 7% increase in secretion of Aβ_x-42_, but not Aβ_x-40_, following 3 minutes of incubation (*p = *0.048). Releasates were also analyzed for Aβ_x-42_/Aβ_x-40 _ratio (Figure 3c) and a significant increase in Aβ_x-42_/Aβ_x-40 _ratio was observed in only one condition: i.e., Aβ_x-42_/Aβ_x-40 _ratio increased by ~4.1% following 1 minute incubation with 10 μM latrepirdine (*p *= 0.024).

### Acute (single dose) of latrepirdine increases ISF Aβ_x-40 _levels in the hippocampus of freely moving mice

**Figure 4:**
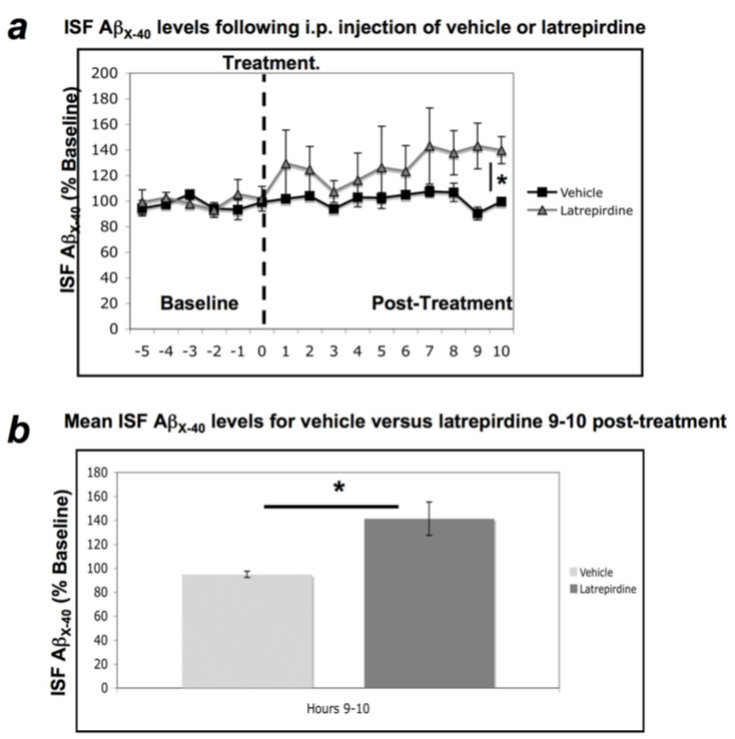
**Acute administration of latrepirdine increases ISF Aβ_x-40 _levels 9-10 hours following treatment**. A single i.p. treatment of latrepirdine (*SD *= 30.94, t(4) = 2.997, *p *= 0.04), but not vehicle (*SD *= 5.68, t(4) = 0.775, *p *= 0.48), produces an increase in ISF Aβ_x-40 _levels from baseline at 9-10 hours post-treatment (*a*). This increase in ISF Aβ_x-40 _level was also determined to be significantly higher than ISF Aβ_x-40 _levels observed in vehicle treated animals at 9-10 hours post-treatment (*SD = *30.94, t(5) = 3.31, *p *= 0.01; *b*). Data are represented as mean percent of baseline +/- S.E.M. *Value represents a significant difference as determined by paired samples t-test (*a*) or independent samples t-test (*b*), two-tailed α = 0.05, *p < 0.05.

Brain ISF soluble Aβ_x-40 _levels in the hippocampus of freely moving Tg2576 mice expressing human Swedish APP were measured by *in vivo *microdialysis using a 38 kDa molecular weight cut-off probe membrane, as previously described [30-32]. ISF samples were collected every 60 minutes for 6 hours prior (baseline) to intraperitoneal (i.p.) injection of either 0.9% saline (vehicle, n = 5) or 3.5 mg/kg latrepirdine (n = 5), and for 10 hours following injection (post-treatment period) and analyzed for Aβ_x-40 _content by sandwich ELISA, as previously described [30-33]. This dose of latrepirdine is comparable to that of a single dose used in the clinical trial of AD patients [11] after normalization to the body surface area of mice. Baseline ISF Aβ_x-40 _levels were averaged for each mouse and an independent samples t-test confirmed that there was no significant difference in baseline ISF Aβ_x-40 _levels for vehicle versus control mice (data not shown). Each time point was normalized to reflect percent baseline for each mouse, and paired samples t-tests reflected significant 41% increase in Aβ_x-40 _levels following treatment with latrepirdine (*p = *0.04), but not for vehicle (~5% decrease; *p *= 0.48). The elevation lasted for a mean of 9-10 hours post-treatment (Figure 4a). An independent samples t-test of mean latrepirdine versus vehicle ISF Aβ_x-40 _levels from 9-10 hours post-treatment confirmed a significant increase in ISF Aβ_x-40 _levels for latrepirdine compared to vehicle (41% increase versus 5% decrease, respectively; *p = *0.01, Figure 4b).

## Conclusions

In this report, we show in three systems that latrepirdine significantly increases extracellular Aβ levels ranging from 5-65% depending on the system and time course of study. While the mechanism of this effect is not entirely clear, latrepirdine has been reported to modulate a variety of neurotransmitter systems [12-17]. Given the apparent benefit of latrepirdine in two neurodegenerative diseases of disparate pathogenesis (i.e., AD and HD), latrepirdine-induced changes in neurotransmission, coupled with altered synaptic activity, may account for the rapid changes in Aβ levels we observed. Whether these rapid latrepirdine-induced changes in soluble Aβ levels feed back to influence synaptic function was not assessed in these studies, nor did we study the long-term effects on Aβ metabolism of latrepirdine over days to months. Thus, it is unknown whether latrepirdine would effect Aβ accumulation in the brain over longer periods of time.

Examples of rapid increases in Aβ release via different mechanisms have been described, e.g. insulin application to cultured neurons stimulates Aβ secretion [23]. Therefore, one speculation is that insulin and sensitizers, which have been shown to be clinically beneficial for treatment of AD [34], might alter growth factor signaling to influence APP processing or synaptic activity [28] to result in Aβ release. Here, we report stimulation of Aβ secretion by yet another clinically beneficial drug (latrepirdine), through a yet unknown mechanism, which may be regulated by synaptic activity (Figures 3a and 3b).

Recent evidence demonstrates that synaptic activity can dynamically alter ISF Aβ levels *in vivo *[30]. Further support for regulation of APP metabolism and Aβ secretion by synaptic activity was recently published, indicating that synaptic activity protects against Aβ-related synaptic alterations by promoting transport of APP to synapses and secretion of Aβ at the synapse, reducing intraneuronal Aβ [28]. Aβ was also recently shown to act as a positive endogenous regulator of release probability at hippocampal synapses [35]. It is also worth noting that Aβ dynamically fluctuates in the ISF of humans recovering from brain injury [33]. In these situations, clinical trends toward recovery of normal, conscious cerebral activity are consistently associated with elevations of ISF Aβ levels [33,36]. Consistent with this model are data indicating that there are diurnal variations in Aβ metabolism, with Aβ levels peaking during wakefulness and declining during sleep [37]. Arancio and colleagues have demonstrated that these physiological picomolar concentrations of Aβ are actually *essential *for long-term potentiation (LTP) formation, and only with nanomolar concentrations does Aβ interfere with LTP [38].

A recent report indicates that latrepirdine inhibits aggregation of TDP-43 (~40% inhibition at 5 μM) in a cellular model of TDP-43 proteinopathy [39], suggesting that latrepirdine may also offer anti-oligomerization properties. High-molecular weight soluble oligomeric (HMW s.o.)Aβ species have been previously shown to produce AD-like cognitive deficits *in vivo *[2], and clearance of HMW s.o.Aβ ameliorated these deficits [2-4]. The reported benefits in AD and HD associated with latrepirdine treatment may be related to anti-oligomerization properties, though this question was not investigated here. Further investigation is necessary to determine whether latrepirdine disrupts the formation of HMW s.o.Aβ species.

In regard to the recently published effects of latrepirdine enhancing cognition in AD [11,13] and HD patients, it is possible that the observed effects are due to its effects on neurotransmitter receptors in the CNS [16,17]. Likewise, the effects we are observing on Aβ metabolism may also be related to effects on neurotransmitter receptors. The relationship between latrepirdine and the typical neurotransmitter replacement therapy associated with AD (i.e. cholinesterase inhibition) is also unknown, although the side effect profile of latrepirdine [11] is not consistent with cholinergic stimulation [40]. Whether the effects of latrepirdine on cognition and Aβ levels are directly related or are coincident is unknown. Further studies are required to determine the long-term effects of latrepirdine on Aβ and the mechanistic link, if any, with the observed human cognitive benefit.

## Methods

### Preparation and handling of latrepirdine

Latrepirdine was synthesized and characterized by SinoChemexper (Shanghai, China) or Nanosyn Inc (Menlo Park, CA; characterization previously [16]) and no significant differences in effects were observed between the two compounds (data not shown). 1D proton NMR spectra of both compounds are shown in Figure 1. Latrepirdine synthesized by Nanosyn Inc and SinoChemexper were determined to be 97.3% and >99.0% pure, respectively. Latrepirdine was freshly dissolved into serum/antibiotic-free high glucose DMEM (DMEM; Gibco) to 1 mM stock and diluted to experimental concentrations in DMEM for cell culture experiments. For synaptoneurosome experiments, latrepirdine was dissolved into dimethylsulfoxide to 10 mM stock and diluted to experimental concentrations in ion-balanced homogenizing buffer [50 mM Hepes, pH 7.5, 125 mM NaCl, 100 mM sucrose, 2 mM potassium acetate and protease inhibitor cocktail (Pierce)]. For *in vivo *experiments, latrepirdine was dissolved into 0.9% saline (vehicle) at a final concentration of 3.5 mg/ml (prepared fresh every 2 days).

### Cell culture experiments

Mouse N2a neuroblastoma cells stably overexpressing human Swedish (K670N, M671L) APP [25] were the generous gift of Dr. Gopal Thinakaran (University of Chicago). Cell were cultured to ~80% confluency in growth media [DMEM, 10% FBS (Gibco), 1% Pen-Strep (Gibco), 0.2 mg/ml G418 (Sigma)] prior to treatment with drug. Cells were washed 1× with ice cold PBS (pH 7.4) then incubated with either latrepirdine or vehicle (DMEM). Conditioned media was collected following 6 hours of treatment, cells were washed 1× with ice cold PBS, and cells were lysed and collected in 1× lysis buffer [20 mM Tris, 137 mM NaCl, 1 mM EDTA, 1 mM pepstatin, 1 mM PMSF, 5 mM ZnCl_2_, 1% Triton X-100, EDTA-free mini-complete protease inhibitor cocktail tablet (Roche)] then centrifuged (14,000 RPM) for 15 minutes at 4°C. Following BCA protein estimation assay (Pierce), 30 μg total protein from conditioned media or 15 μg total protein from cell lysates was loaded onto a 12% Bis-Tris (Bio Rad) gel for SDS-PAGE with NuPage MES buffer (Invitrogen) at 200 V for 35 minutes, then transferred to PVDF membrane (0.22 μm for conditioned media or 0.45 μm for cell lysates; Millipore) using the Trans-Blot SD transfer apparatus (Bio-Rad). Following transfer, the membrane was boiled for 5 minutes in 1× PBS, blocked for 1 hour in 5% non-fat milk (Santa Cruz Biotechnology), and incubated with either mouse mAb 6E10 (Covance, Aβ_1-16_), rabbit pAb G369 (APP C-terminus), or mouse mAb anti-Actin (Sigma) overnight at 4°C. Membranes were washed 4×, incubated in HRP-conjugated goat anti-mouse or anti-rabbit IgG secondary antibody (Vector Laboratories), washed 4×, and developed with ECL western blotting substrate (Pierce) using the Fujifilm LAS-3000 developer. Band intensity was measured with MultiGauge (Fujifilm) software and normalized to percent of vehicle. For holoAPP and APP CTFs, band density was normalized first to Actin band density (to control for any loading error), prior to comparison to vehicle. Total Aβ and sAPPα levels were compared from conditioned media by western blot analysis. Conditioned media was also analyzed for secreted Aβ_x-42_/Aβ_x-40 _ratio by sandwich ELISA (Wako) according to manufacturer protocols.

### Cortical synaptoneurosome preparation from TgCRND8 mice

Synaptoneurosomes were prepared from the cortices of postnatal day (P)10-14 TgCRND8 (APP K670N, M671L, V717F) [27] mice. Briefly, mice were quickly decapitated, brains were removed and dissected, and cortices were homogenized in a glass-Teflon homogenizer in homogenization buffer (HB), filtered through a series of nylon mesh filters (149, 62, and 30 microns; Small Parts) and then through a 10- μm polypropylene filter (Gelman Sciences) [29]. Filters were washed at each step with HB. The final filtrate was spun briefly (4,000 × g, 1 min), then the supernatant was spun (7,000 × g, 15 min) to pellet synaptoneurosomes. Synaptoneurosomes were resuspended in the fresh HB. Before drug treatment paradigms, this suspension was incubated on ice while stirring, with 1 μM tetrodotoxin (Tocris), for 5 min then at room temperature for another 5 min. Reactions proceeded at room temperature. Samples were removed and instantly put on ice at 0 (immediately before adding latrepirdine), 1, 3, 5, and 10 minutes following latrepirdine application. Each sample was spun (20,000 × g, 10 min) and the supernatant was collected as releasate using protein LoBind tubes (Eppendorf). Samples were analyzed for secreted Aβ_x-40 _and Aβ_x-42 _by sandwich ELISA (Wako) according to manufacturer protocols.

### In vivo microdialysis of hippocampal interstitial fluid (ISF) Aβ_x-40 _levels

Soluble Aβ levels were measured from the brains of 3-month old Tg2576 hemizygous (Swedish APP) mice as described previously [30-32]. Briefly, Tg2576 mice received a single dose of 3.5 mg/kg latrepirdine (n = 5) or equivalent volume of vehicle (n = 5) by intraperitoneal (i.p.) injection. *In vivo *microdialysis was used to assess hippocampal ISF levels of Aβ_x-40 _from awake, freely moving Tg2576 mice (as described [32]). Samples were collected (by refrigerated fraction collector) every 60 minutes for 6 hours prior to injection (baseline) and 10 hours following injection of drug or vehicle. Aβ_x-40 _levels were assessed by sandwich ELISA following completion of the experiment (described previously in [30,32]). During microdialysis, awake and freely moving mice were housed in constant light with *ad libitum *access to food and water.

### Statistical analysis

For quantification of total holoAPP, APP-CTFs, Aβ and sAPPα from cell lysates and conditioned media, independent samples t-tests were utilized to determine significant mean differences for each condition versus control. For synaptoneurosome time-course experiments, paired samples t-tests were used to determine pre-treatment versus post-treatment differences of secreted Aβ_x-40 _and Aβ_x-42 _levels, and for Aβ_x-42_/Aβ_x-40 _ratios. For microdialysis time-course experiments, 6 pre-treatment ISF samples were collected and analyzed for Aβ_x-40 _levels, averaged for each animal to produce a baseline value of ISF Aβ_x-40 _levels, and each time point was normalized to percent of baseline for each animal. Paired samples t-test was used to determine baseline versus 9-10 hour post-treatment differences for ISF Aβ_x-40 _levels and independent samples t-tests were utilized to compare mean post-treatment ISF Aβ_x-40 _levels for vehicle versus latrepirdine. Significance for t-tests are reported with a p < 0.05 using two-tailed tests with an α-level of 0.05.

## Competing interests

MS: Medivation (Consultant for clinical development of latrepirdine); MEE: Medivation (Grantee for study of latrepirdine mechanisms of action in Huntington's Disease); SG: J&J/Elan (Safety Monitoring Committee); Diagenic, Amicus (Consulting); Forest, Amicus (Grantee).

## Authors' contributions

JWS and SG prepared the manuscript. JWS contributed cell culture experiments, SHK contributed synaptoneurosome experiments, and JRC, DKV, and JLR contributed in vivo microdialysis experiments. MEE, MS, IB and DMH contributed critical reading of the manuscript and experimental design. Development and characterization of the TgCRND8 mouse model was contributed by DW, PF and PSH. SG had full access to all of the data in the study and takes responsibility for the integrity of the data and the accuracy of the data analysis. All authors have read and approved the final manuscript.
